# Montreal Veterinary Medical Association

**Published:** 1896-02

**Authors:** Harri H. Dell

**Affiliations:** Secretary-Treasurer


### SOCIETY PROCEEDINGS.

### MONTREAL VETERINARY MEDICAL ASSOCIATION.

THIS Association met in the Library of the Faculty of Comparative Medicine and Veterinary Science on Thursday evening, November 7, with the President, Dr. M. C. Baker, in the chair. Drs. McEachran, Adami, Mills, and Martin were also present.

The minutes of the last meeting were read and approved. The Secretary was then instructed to convey the thanks of the members to Dr. F. H. Osgood, Chairman of the Massachusetts State Board of Cattle Commissioners, for the donation of fifty copies of the report of the Board for 1895.

Dr. D. McEachran reported the addition to the library of Moller's *Surgery*, and also Vol. II. of Fleming's *Surgery*.

Mr. Thurston reported a case of "Obstruction in the Thoracic Portion of the Œsophagus of a Dog, Due to the Presence of a Bone,"[1] with symptoms of gastro-intestinal irritation, the animal dying shortly after admission to the hospital. The post-mortem revealed an ulcerative condition and a perforation through the œsophageal tissue into the aorta, death being due to hemorrhage into the stomach through this aperture. Drs. McEachran and Mills reported some similar cases from their own experience.

Mr. Harri H. Dell presented a carefully prepared paper on "Pyæmia in the Dog," and illustrated his remarks by microscopic and macroscopic specimens from a case which recently came under his observation. In the discussion following Dr. Adami congratulated the essayist on his contribution to our literature on the subject. He also referred to the experiments of Councilman, which demonstrated the fact that pus can be produced by caustic substances without the intervention of pyogenic organisms.

Dr. McEachran gave some further remarks on the history of the disease, and instanced the occurrence of empyema in a cow following mammitis.

Dr. Mills referred to the literary and scientific merit of the essay and reported the occurrence of the disease in his own kennel.

Dr. Martin followed with very interesting remarks on the pathology of pyæmia and septicæmia.

The essayists for the next meeting were then appointed, and the meeting adjourned.

A REGULAR meeting of the Association was held on Thursday evening, November 21st, 1895, with the Honorary President, Dr. D. McEachran, in the chair.

The minutes of the previous meeting having been read and adopted, a case reported by Mr. J. S. Patterson was presented for diagnosis.

The animal in question was a bay horse, five years old, weight 1000 pounds. When called to see the case found the animal in a recumbent position, unable to rise. Temperature 100.5°, pulse 46, respiration 12.

[1] Reported in this number. See p. 130.

When assisted to his feet moved without difficulty and seemingly all right. The next day he was down again; was again raised and placed in slings, and received a laxative, followed by nerve tonics.

In twenty-one days was sent home with no abatement of the symptoms. Was put to work, which he performed satisfactorily, but every morning had to be assisted to his feet. Some three months after he fell on the pavement, and, being unable to rise, was shot by a policeman. No post-mortem was held, but it was the consensus of opinion of those present that the difficulty in rising was due to anchylosis of some of the vertebræ.

Mr. John Greer contributed a lengthy, but carefully prepared, paper on diagnosis and general symptoms of disease.

A prolonged discussion ensued on the subject of the variation of the pulse in different animals and in those of the same species.

Dr. D. McEachran addressed the meeting on the subject of "Coughs as Symptomatic of Disease with Special Reference to the Horse."

Dr. M. A. Dawes, of the Health Department, followed with some good advice to the experiment committee.

The essayists for the next meeting were then notified, and the meeting adjourned.

A REGULAR meeting of the above-named Association was held in the Library on December 5, 1895, the President, Dr. M. C. Baker, occupying the chair. The roll-call showed a good attendance of members and visitors. The minutes of the previous meeting were read and approved.

A report was received from the experimental committee on some experiments with pilocarpin and eserine, the combination not having proven satisfactory in the cases under observation.

Mr. Charles H. Higgins, B.S., presented a paper on "Bacteriology and its Practical Applications."

He gave an extended historical résumé of the development of bacteriological science, which development was largely due to the discovery and subsequent improvement of the microscope. The theories of immunity advanced by various investigators were each in turn enumerated, and the fallacies of several were clearly demonstrated. Many interesting topics arose out of the discussion which followed, among them being inspection of milk and propagation of tuberculosis; tuberculin and mallein and the dosage of each.

Mr. John Greer reported a case of "Septic Aspiration-Pneumonia in a Cow." The first symptom noticed in the animal was diminished lactation. Soon a swelling appeared on the left cheek over the region where Steno's duct discharges into the buccal cavity. This swelling increased until there was occlusion of the lachrymal duct and a bulging of the eye from the orbit. Actinomycosis was suspected, but microscopic examination of the purulent matter in the swelling failed to verify the suspicion. Febrile symptoms developed, accompanied by chills, weak pulse, depression, and diarrhœa, death occurring about four weeks after the first appearance of the swelling. The post mortem revealed a large abscess on the inferior maxilla with softening and necrosis of the adjacent tissues. The lungs contained multiple abscesses, due to the inspiration of septic germs from the primary abscess of the jaw. A pneumonia had developed in addition. The term septic aspiration-pneumonia was very appropriate. In Mr. Greer's opinion, which was concurred with by those present, had the original abscess been recognized early and subjected to proper treatment, the animal would have made a good recovery. The case was an interesting one, and gave rise to considerable discussion.

The essayist for the next meeting was appointed, also a member to serve on the experimental committee.

HARRI H. DELL,
Secretary-Treasurer.

